# Comparison of self-reports and biomedical measurements on hypertension and diabetes among older adults in China

**DOI:** 10.1186/s12889-020-09770-7

**Published:** 2020-11-07

**Authors:** Donghong Xie, Jiwen Wang

**Affiliations:** School of Sociology and Population Studies, Renmin University of China, Beijing, China

**Keywords:** Self-reporting, Biomedical test, Discrepancy, Sociodemographic characteristics

## Abstract

**Background:**

Researchers interested in the effects of health on various life outcomes often use self-reported health and disease as an indicator of true, underlying health status. However, the validity of reporting is questionable as it relies on the awareness, recall bias and social desirability. Accordingly, biomedical test is generally regarded as a more precise indication of the disease.

**Methods:**

Using data from the third wave of China Health and Retirement Longitudinal Study (CHARLS), we selected individuals aged 40–85 years old who participated in both health interview survey and biomedical test. Sensitivity, specificity, false negative reporting and false positive reporting were used as measurements of (dis) agreement or (in) validity, and binary and multinomial logistic regression were used to estimate under-report or over-report of hypertension and diabetes.

**Results:**

Self-reported hypertension and diabetes showed low sensitivity (73.24 and 49.21%, respectively) but high specificity (93.61 and 98.05%, respectively). False positive reporting of hypertension and diabetes were 3.97 and 1.67%, while false negative reports were extremely high at 10.14 and 7.38%. Educational attainment, *hukou*, age and gender affected both group-specific error and overall error with some differences in their magnitude and directions.

**Conclusion:**

Self-reported conditions underestimate the disease burden of hypertension and diabetes in China. Adding objective measurements into social survey could improve data accuracy and allow better understanding of socioeconomic inequalities in health. Furthermore, there is an urgent need to provide basic health education and physical examination to citizens, and promote the use of healthcare to lower the incidence and unawareness of disease in China.

## Background

Hypertension and diabetes are two well-known risk factors of cardiovascular disease, the leading cause of death worldwide with 17.79 million deaths in 2017 [1]. High-quality estimates of prevalence based on biomedical measurements are needed for monitoring cardiovascular disease risks and planning public health preventions and interventions. Due to the high cost and long-time collection of biomedical data, economists and demographers have relied heavily on self-reported hypertension and diabetes to estimate their prevalence and disease burden. However, recent research has raised doubts about the reporting error of self-reported disease [2, 3].

Many a study has attempted to assess the value of a self-reported disease by comparing self-reports with objective assessments, which has many advantages: precision of measurement, reliability, and less bias than questionnaires [4]. The criterion shows the discrepancy between self-reports and biomedical tests and is usually measured by sensitivity and specificity of the errors in a given group, as well as false reporting of the overall error. Sensitivity was defined as the percentage of respondents who reported hypertension/diabetes among those with biomedical hypertension/diabetes. This value was thus equivalent to hypertension/diabetes awareness among those with diseases; specificity was defined as the percentage of individuals who reported no hypertension/ diabetes among those with ‘normal’ biomedical measurements; false negative reporting was defined as those who reported no hypertension/diabetes but were diagnosed hypertension/diabetes, and false positive reporting was defined as those who reported hypertension/diabetes but were not diagnosed hypertension/diabetes. Evidence from developed countries indicated that there was a big gap between self-reported diseases and biomedical diseases of hypertension and diabetes and it may differ by socioeconomic groups. More educated and higher socioeconomic individuals might have a better understanding of health information and were more capable of answering survey questions on disease diagnosis [5–8].

Although developed countries have witnessed substantial disagreements between self-reports and objective diagnosis of the two diseases, the performance of developing countries were unclear due to the limited data. A handful of studies have investigated the incidence of disease in some local areas [9–11], but there is still a debate regarding the consistency and direction of the social gradient and health in developing countries [12], which means socioeconomic status might differ in developing countries compared to developed contexts. One possible explanation is that economic development takes place unequally across regions, and so does progress in the awareness of a disease. In addition, populations are exposed to different health-enhancing or health-damaging conditions. For example, the household registration (*hukou*) system divided China into two separated societies, with the majority of the population confined in the rural areas and entitled to fewer rights and benefits compared with urban residents.

With approximately 20% of the world’s population, China is experiencing rapid population aging and an epidemiological transition moving from the primacy of acute, infectious and deficiency diseases to the increasing dominance of non-communicable and chronic conditions [12]. Report on Nutrition and Chronic Diseases of Chinese Residents (2015) shows that the prevalence of hypertension and diabetes in China has reached 25.2 and 9.7% over 18 years old, respectively. The lack of medical insurance and the high cost of the health care market may lead to incorrect perception of disease prevalence, which means under-diagnosis of chronic disease is not uncommon in China.

However, whether and to what extent the self-reported and biological hypertension and diabetes conflict is not clear in China. Meanwhile, diabetes mellitus causing the most death rises from the 19th in 1990 to 8th in 2017 and high systolic blood pressure was the leading risk factors contributing to deaths and Disability-Adjusted Life Year (DALY) in China [13]. Therefore, understanding the reality of the two diseases, estimating the burden of diseases and intervening the risk of death from diseases are very important in China.

## Methods

### Study design and setting

Data for this analysis are from China Health and Retirement Longitudinal Study (CHARLS), which is a nationally representative survey in China, designed by the National School for Development (China Center for Economic Research) together with the Institute for Social Science Survey at Peking University. The baseline wave of CHARLS was being fielded in 2011 and included about 10,000 households and 17,500 individuals in 150 counties/districts and 450 communities. The multistage sample was drawn at each stage based on probability proportional-to-size random-sampling procedures. The survey collected detailed demographic background, socioeconomic information, health status and functioning. The analysis draws on data of Wave 3 (2015), because it collected reliable venous blood samples which allow us to estimate the discrepancy between self-reported diseases and underlying biomarker levels among CHARLS respondents. Detailed information about the CHARLS blood sampling procedure and data quality management has been published previously [14]. All participants signed informed consent, and CHARLS was approved by the Ethical Review Committee of Peking University.

We restricted respondents aged 40 to 85 years old with valid self-reported diseases and biomedical tests. The Wave 3 (2015) included 20,284 respondents asked to consent to a venous blood draw and blood pressure measurement; 13,013 respondents provided venous blood and 16,406 respondents provided blood pressure information. Combined with those respondents who also provided detailed sociodemographic information, the final sample sizes of hypertension and diabetes were 14,462 and 12,189, respectively. Characteristics of the sample are summarized in Table 1.^1^

**Table 1:** Characteristics of the sample, China Health and Retirement Longitudinal Study

Variable	Total	Hypertension	Diabetes
	(***N*** = 19,292)	(***N*** = 14,462)	(***N*** = 12,189)
**Education**			
Illiterate	24.4% (4707)	25.4% (3673)	25.6% (3120)
Primary education	45.4% (8759)	46.2% (6681)	45.9% (5595)
Secondary education and above	30.2% (5826)	28.4% (4107)	28.5% (3474)
***Hukou***			
Urban	23.0% (4437)	20.3% (2936)	20.3% (2474)
Rural	77.0% (14855)	79.7% (11526)	79.7% (9715)
**Drinking**			
None	73.2% (14122)	73.6% (10644)	73.8% (8995)
Less than 3 days a month	6.2% (1196)	6.1% (882)	5.9% (719)
Once or 2 to3 days a week	6.4% (1235)	6.3% (911)	6.2% (756)
4 to 6 days a week or daily	8.8% (1698)	8.5% (1229)	8.5% (1036)
Twice a day or above	5.4% (1042)	5.5% (795)	5.6% (683)
**Number of cigarettes/days**	1.7 (19292)	1.7 (14462)	1.7 (12189)
**Sex**			
Female	52.3% (10090)	53.5% (7737)	54.0% (6582)
Male	47.7% (9202)	46.5% (6725)	46.0% (5607)
**Age**			
40–49	21.5% (4148)	19.5% (2820)	18.6% (2267)
50–59	32.1% (6193)	31.8% (4599)	32.0% (3900)
60–69	29.8% (5749)	31.5% (4556)	32.4% (3949)
70–79	13.6% (2624)	14.3% (2068)	14.4% (1755)
80 and above	3.0% (579)	2.9% (419)	2.6% (317)
**Marriage**			
Unmarried	12.2% (2354)	12.2% (1764)	12.2% (1487)
Married	87.8% (16938)	87.8% (12698)	87.8% (10702)

### Measurement

#### Self-reports and biomedical measurements of hypertension and diabetes

Self-reported data on hypertension and diabetes were obtained by the question, “Have you been diagnosed with [conditions listed below, read one by one] by a doctor?”, and there are 14 options, which are “Hypertension, Dyslipidemia, Diabetes, Cancer or malignant tumor, Chronic lung diseases, Liver disease, Stroke, Heart problems, Kidney disease, Stomach or other digestive disease, Emotional, nervous, or psychiatric problems, Memory-related disease, Arthritis or rheumatism and Asthma”.^2^ If a respondent answered hypertension or diabetes, we defined the self-reported hypertension or diabetes as 1, otherwise as 0. Biomedical blood pressure was measured three times (approximately 45 s apart) on a single occasion, using an electronic monitor. We take the average of the last 2 readings, after excluding the first reading to avoid white coat hypertension. Hypertension was defined as a systolic blood pressure ≥ 140 mmHg and/or a diastolic blood pressure ≥ 90 mmHg and/or current use of antihypertensive medication, following the WHO guideline [15]; biomedical diabetes was measured by venous blood data which provided glycated hemoglobin (HbA1c). The diagnostic criterion for diabetes in our study was defined as HbA1c values ≥6.5%. If a respondent’s glycated hemoglobin was over 6.5%, we defined the biomedical diabetes as 1, otherwise as 0. HbA1c is more expensive than the routinely conducted test, and may not be the most widely used screening test, however, it can be measured at any time of the day regardless of the duration of fasting or the content of the previous meal and it is a good predictor for both the micro- and macrovascular complications of diabetes. The additional benefits in predicting costly preventable clinical complications may make it a cost-effective choice [16].

#### Economic resources

Educational attainment was measured by three levels. This variable indicated the highest educational degree attained by respondents at the survey point. The lowest category was individuals holding no formal education (illiterate); intermediate education ranged from not finishing primary school, home school to elementary school; and the highest category was respondents holding middle school or above degree. *Hukou* was measured by whether the respondent held a rural *hukou* (0 = No, 1 = Yes).

#### Health behaviors

Drinking was a 5-category variable indicating the frequency of drinking last year: none (coded as 1), less than 3 days/month (coded as 2), less than 3 days/week (coded as 3), 4 to 6 days/week or daily (coded as 4), twice a day or above (coded as 5). Smoking was a continuous variable indicating the number of cigarettes/day, which ranged from 0 to 100.

#### Demographic characteristics

Gender was a binary variable: male (coded as 1), female (coded as 0). Age was a 5-category variable ranging from 40 to 49, 50–59, 60 to 69, 70–79, 80 and above. Marital status indicated whether the respondent was in a marriage status: separated, divorced, widowed and never married (coded as 0), married or partnered (coded as 1).

### Analytic strategy

Our first step was to assess the difference in prevalence estimates based on two data collection methods, the prevalence of hypertension and diabetes were calculated according to self-reported information, as well as according to the results of biomedical measurements obtained from the CHARLS. We use the formula to calculate the degree of underestimation as follows.

$$ \mu =\frac{\mathrm{Biomedical}\ \mathrm{test}-\mathrm{Self}-\mathrm{reports}}{\mathrm{Biomedical}\ \mathrm{test}}\ast 100\% $$

To assess the accuracy of self-reported data, sensitivity, specificity, false negative reporting and false positive reporting were also calculated, respectively. Only sensitivity or specificity was of no practical use when it came to helping the clinician estimate the probability of disease in individual patients [17]. In addition, sensitivity and specificity assessed group-specific errors in diagnosed or undiagnosed diseases, respectively, but not overall errors. We identified both overall error and the group-specific error and assessed sociodemographic characteristics that are correlated with misreporting (sensitivity, specificity, false negative reporting and false positive reporting). Controlling for educational attainment, *hukou*, drinking, number of cigarettes/day, age, gender and marital status, binary and multinomial logistic regression analysis were applied. As the overall error outcome (correct reporting, false negative reporting and false positive reporting) has more than two categories. The model equations are set as follows^3^:

$$ Ln\left(\frac{\Pr \left( Yi= False\ negative\ reporting\right)}{\Pr \left( Yi= Correct\ reroptin\mathrm{g}\right)}\right)={\beta}_0+{\beta}_1 Economics+{\beta}_2 Behaviors+{\beta}_3 Demographics+\dots +\varepsilon $$

$$ Ln\left(\frac{\Pr \left( Yi= False\ positive\ reporting\right)}{\Pr \left( Yi= Correct\ reropting\right)}\right)={\beta}_0+{\beta}_1 Economics+{\beta}_2 Behaviors+{\beta}_3 Demographics+\dots +\varepsilon $$

## Results

### Sensitivity, specificity and false reporting of hypertension and diabetes

The prevalence of hypertension was 37.88% based on biomedical test and 31.72% based on self-reported data, indicating that self-reporting led to an underestimation of hypertension by 16.26%. Likewise, the prevalence of diabetes was 14.52% based on biomedical test and 8.81% based on self-reports, indicating an underestimated prevalence of diabetes by 39.32%.

**Fig. 1:**
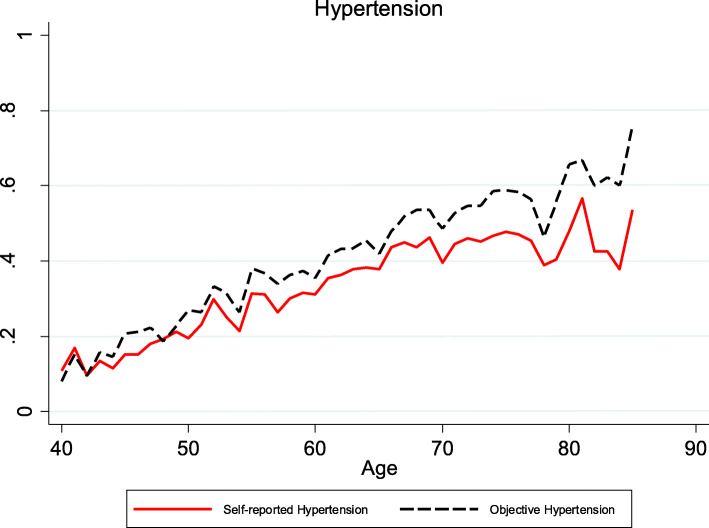
Self-reported hypertension and biomedical hypertension by age

**Fig. 2:**
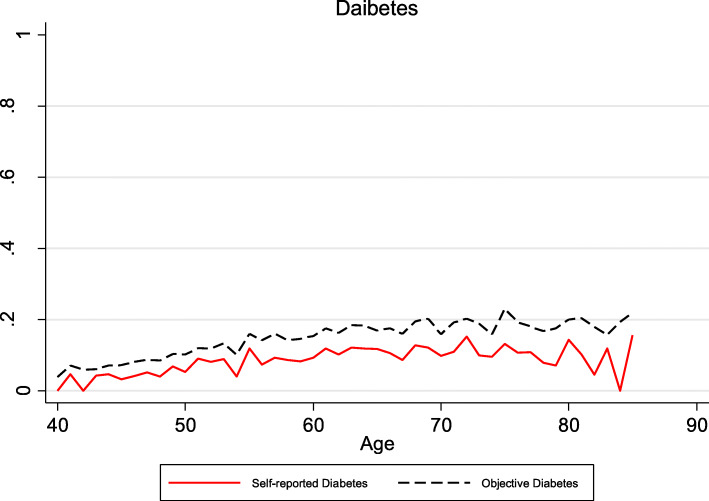
Self-reported diabetes and biomedical diabetes by age

Both the prevalence of self-reports and biomedical hypertension and diabetes increased with age in China, however, biomedical hypertension and diabetes rose considerably faster with age than self-reporting, which meant the discrepancy between self-reports and biomedical hypertension increased with age as was shown in Figs. 1 and 2. This is suggestive of undiagnosed hypertension and diabetes becoming more of a problem increasing with individuals’ age. Since China has the largest number of older adults in the world according to United Nations data, and undiagnosed high blood pressure and diabetes may become increasingly common over time.

Table 2 provides the sensitivity, specificity and false reporting of self-reported hypertension and diabetes compared with biomedical tests. The overall sensitivity and specificity of self-reported hypertension were 73.24 and 93.61%, which meant 26.76% of respondents didn’t know they had hypertension, and 6.39% of respondents falsely thought they had hypertension. The false reporting of hypertension was 14.11%: specifically false positive reporting of hypertension was 3.97% and false negative reporting was 10.14%. For diabetes, the overall sensitivity and specificity were 49.21 and 98.05%, which meant over 50% of respondents didn’t know they had hypertension, and only less than 2% of respondents falsely thought they had diabetes. The false reporting of diabetes was 9.05%: specifically false positive reporting of hypertension was 1.67% and false negative reporting was 7.38%, which meant about 10% of respondents misreport diabetes status.

**Table 2:** Sensitivity, specificity, false negative reporting and false positive reporting (%)

	Hypertension	Diabetes
Sensitivity	73.24	49.21
Specificity	93.61	98.05
False negative reporting	10.14	7.38
False positive reporting	3.97	1.67

Comparing the four indicators above of hypertension and diabetes, we found that the overall misreporting error is different from the group-specific error. Taken together, these results were suggestive of a substantial public health problem of undiagnosed hypertension and diabetes in China. Self-reported hypertension and diabetes increase the risk of underdiagnosis and underestimate true disease burden in the population substantially.

### Sociodemographic characteristics of sensitivity, specificity and false reporting

The estimated effects on our two sets of the discrepancy between self-reported hypertension/diabetes and their biomedical tests using binary and multinomial logistic regression were shown in Tables 3 and 4. Columns 1, 2 and 3 showed the estimated effects of predictor variables on sensitivity and specificity between self-reported conditions and their biomedical tests, and correct reporting using binary logistic regression models. Columns 4 and 5 showed the estimated effects of predictor variables on false negative and positive reporting using multinomial logistic regression models.

**Table 3:** Predicting disagreement or false reporting of hypertension

	Sensitivity	Specificity	Correct Reporting	False Negative VS Correct Reporting	False Positive VS Correct Reporting
Hypertension					
Education (Illiterate = 0)					
*Primary education*	1.141	0.910	1.081	0.868^+^	1.097
	(1.629)	(−0.803)	(1.224)	(−1.951)	(0.791)
*Secondary and above*	1.200^+^	0.857	1.083	0.843^+^	1.164
	(1.860)	(−1.125)	(1.054)	(−1.949)	(1.114)
*Hukou* (Urban = 0)	0.735^***^	1.023	0.896^+^	1.135^+^	1.069
	(−3.773)	(0.195)	(−1.705)	(1.683)	(0.596)
Drink (None = 0)					
*< 3 days a /month*	1.051	1.223	1.115	0.920	0.846
	(0.341)	(1.017)	(0.975)	(−0.644)	(−0.850)
*Once or 2 to 3 days a/week*	0.908	0.847	0.823^+^	1.239^+^	1.162
	(−0.734)	(− 0.937)	(−1.951)	(1.856)	(0.855)
*4 to 6 days a/week or daily*	0.628^***^	0.697^*^	0.640^***^	1.613^***^	1.443^*^
	(−4.235)	(−2.397)	(−5.394)	(5.060)	(2.459)
*Twice a day or above*	0.599^***^	0.962	0.692^***^	1.585^***^	1.070
	(−3.982)	(−0.191)	(−3.673)	(4.144)	(0.337)
Number of cigarettes/day	0.927^**^	1.105^*^	1.015	1.004	0.936
	(−2.696)	(2.458)	(0.682)	(0.166)	(−1.640)
Sex (female = 0)	0.829^*^	0.847	0.816^**^	1.261^**^	1.153
	(−2.319)	(−1.466)	(−3.213)	(3.161)	(1.273)
Age (40–49 = 0)					
*50–59*	1.372^**^	0.937	0.841^*^	1.359^***^	0.910
	(2.898)	(−0.519)	(−2.279)	(3.337)	(−0.755)
*60–69*	1.948^***^	0.672^**^	0.781^***^	1.429^***^	1.042
	(6.244)	(−3.211)	(−3.301)	(3.929)	(0.334)
*70–79*	1.720^***^	0.731^+^	0.672^***^	1.876^***^	0.830
	(4.553)	(− 1.903)	(−4.484)	(6.071)	(−1.147)
*> =80*	1.222	0.572^+^	0.443^***^	3.032^***^	0.908
	(1.177)	(−1.789)	(−5.852)	(7.217)	(−0.319)
Marriage (Not = 0)	1.038	1.232	1.156^*^	0.864^+^	0.868
	(0.413)	(1.498)	(1.970)	(−1.759)	(−1.032)
*N*	5478	8984	14,462	14,462	14,462

Note: + *p* < 0.10; * *p* < 0.05; ** *p* < 0.01; *** *p* < 0.001; odds ratio estimates, logistic and multinomial logistic models, with z scores in parentheses

**Table 4:** Predicting disagreement or false reporting of diabetes

	Sensitivity	Specificity	Correct Reporting	False Negative VS Correct Reporting	False Positive VS Correct Reporting
Diabetes					
Education (Illiterate = 0)					
*Primary education*	1.136	0.959	1.057	0.930	1.029
	(1.009)	(−0.216)	(0.681)	(− 0.814)	(0.149)
*Secondary and above*	1.436^*^	0.745	1.124	0.811^+^	1.310
	(2.423)	(−1.337)	(1.177)	(−1.916)	(1.233)
*Hukou* (Urban = 0)	0.616^***^	1.420^*^	1.186^*^	0.869	0.752^+^
	(−4.147)	(2.050)	(2.118)	(−1.577)	(−1.669)
Drink (None = 0)					
*< 3 days a /month*	0.942	0.545^*^	0.948	0.892	1.873^*^
	(−0.261)	(−2.311)	(−0.376)	(−0.690)	(2.389)
*Once or 2 to 3 days a/week*	0.918	0.589^+^	0.996	0.864	1.732^*^
	(−0.373)	(−1.938)	(− 0.028)	(− 0.886)	(2.012)
*4 to 6 days a/week or daily*	0.516^**^	0.787	0.872	1.113	1.319
	(−3.224)	(−0.866)	(−1.139)	(0.818)	(1.000)
*Twice a day or above*	0.433^**^	0.886	1.046	0.910	1.200
	(−2.976)	(−0.346)	(0.294)	(−0.564)	(0.521)
Number of cigarettes/day	0.940	1.122	1.040	0.976	0.897
	(−1.292)	(1.577)	(1.268)	(−0.726)	(−1.488)
Sex (female = 0)	1.005	1.337	1.117	0.930	0.752
	(0.042)	(1.543)	(1.315)	(−0.780)	(−1.516)
Age (40–49 = 0)					
*50–59*	1.542^*^	0.892	0.765^*^	1.371^**^	1.072
	(2.394)	(−0.494)	(−2.505)	(2.659)	(0.304)
*60–69*	1.819^***^	0.629^*^	0.614^***^	1.676^***^	1.456^+^
	(3.423)	(−2.074)	(−4.707)	(4.477)	(1.686)
*70–79*	1.430^+^	0.521^*^	0.521^***^	1.967^***^	1.745^*^
	(1.801)	(−2.491)	(−5.446)	(5.102)	(2.133)
*> =80*	1.105	0.440^+^	0.456^***^	2.228^***^	2.068
	(0.308)	(−1.820)	(−3.986)	(3.735)	(1.617)
Marriage (Not = 0)	1.277	0.907	1.059	0.918	1.089
	(1.629)	(−0.431)	(0.600)	(−0.830)	(0.376)
N	1770	10,419	12,189	12,189	12,189

Note: + *p* < 0.10; * *p* < 0.05; ** *p* < 0.01; *** *p* < 0.001; odds ratio estimates, logistic and multinomial logistic models, with z scores in parentheses

#### Hypertension

First, we looked into the group-specific error. Respondent characteristics associated with sensitivity found that individuals with higher educational attainment, urban *hukou*, aged ≥50 years, female, and having healthy lifestyle, were strongly and independently associated with having more accurate self-reported hypertension than their counterparts among those with biomedical hypertension. The results in Table 3 suggested that no respondent characteristic was significantly associated with more accurate reporting in specificity except for age and healthy lifestyle. Generally, older adults were more likely to erroneously report the absence of hypertension than those younger than 60 years old. People who drank alcohol every day were more likely to report errors among those without hypertension, however, those who smoked more frequently were more likely to correctly report the absence of hypertension.

Next, we identified the overall error of hypertension. The likelihood of reporting errors decreased with educational attainment, but not significantly. Urban *hukou*, no drinking, female, younger age and married respondents were strongly and independently associated with correct reporting (Columns 3 in Table 3). Compared with urban *hukou*, rural *hukou* had a 10.4% increase in misreporting. Compared with non-drinkers, people who drank every day or more were more likely to report errors. Males were more likely to erroneously report than females. Older adults were more easily to erroneously report than those younger than 50 years of age, and the error rate increased with age. The unmarried were more prone to misreport than those married.

Educational attainment had a significant effect on the risks of false negative reporting but not significantly on false positive reporting (Columns 4–5 in Table 3). The propensity of false negative reporting went down significantly with educational attainment. Compared with the illiterate, primary education had a 13.2% decrease in the rate of false negative reporting while secondary education and above had a 15.7% decrease. Although people with higher educational attainment reported higher false positive than their counterparts, the effect was not significant. Individuals having a rural *hukou*, aged 50 years old and above, male, and having unhealthy lifestyle, were strongly associated with false negative reporting. False positive reports were almost not related to sociodemographic characteristics.

#### Diabetes

For self-reported diabetes, educational attainment, *hukou*, drinking and age were associated with sensitivity. Aged participants with higher levels of education, having an urban *hukou* and less drinking were also more likely to accurately self-reported diabetes than their counterparts among those with biomedical diabetes (Column 1 in Table 4). Multivariate analyses showed that younger respondents with a rural *hukou* and less drinking had slightly more accurate reporting on the absence of diabetes than their counterparts among those without diabetes (Column 2 in Table 4).

Our indicator of educational attainment had almost no statistically significant effect on correct reporting and false positive reporting of diabetes, except that secondary education and above might slightly reduce false negative reporting (Columns 3–5 of Table 4). Contrary to our expectations, false positive reporting between self-reported diabetes and biomedical diabetes did not depend on educational attainment, which was similar to previous research [18]. Compared with respondents having urban *hukou*, those having rural *hukou* had a 18.6% increase in the rate of correct reporting due to the lower prevalence of diabetes in rural area. Older adults were more prone to false reporting and false negative reporting than those younger than 50 years of age, and the rate went up significantly with age. To be specific, the false negative reporting of the respondents aged 50–59, 60–69, 70–79, 80 and above years old was 37.1, 67.6, 96.7, 122.8% higher than the reference group.

The results suggest that educational attainment, *hukou*, age and gender affect both the group-specific error and the overall error of hypertension and diabetes reporting, but there are some differences in their magnitude and directions.

## Discussion

Using data from Chinese older adults aged over 40 and above, we analyzed two diseases that were commonly used in clinical evaluations of health-related risk, and estimated the discrepancy between self-reports and biomedical measurements. We found a large difference in the respondents who reported having hypertension and diabetes (31.72 and 8.81%) relative to those who were measured to have two diseases (37.88 and 14.52%). Depending on only self-reported disease might underestimate the true extent of the disease burden in China, and self-reporting led to an underestimation of hypertension and diabetes by 16.26 and 39.32%, respectively. Due to the more complex collection methods and higher cost, we find that diabetes is more prone to be underestimated, which is really not conducive to disease assessment and intervention.

We also examined the sociodemographic characteristics correlated with misreporting by using four indicators (sensitivity, specificity, false negative reporting and false positive reporting). For hypertension, we found that educational attainment, *hukou*, age and gender affected both the group-specific error and the overall error, but there were some differences in their magnitude and directions. Educational attainment was an important explanatory factor for sensitivity and false negative reporting, with each additional gradient decreased in education reducing the probability of committing false negative reporting of hypertension, respectively. Besides, *hukou*, as one of the most important redistributive institutions in China, had an effect on sensitivity and false negative reporting: the rural adults were more likely to falsely report among those people with hypertension and have a false negative reporting. For diabetes, educational attainment was also an important explanatory factor for sensitivity and false negative reporting, which meant the least educated people might underestimate their diabetes status than their counterparts. Besides, *hukou* had an effect on sensitivity, specificity and correct reporting: the rural adults were more likely to falsely report among those people with diabetes and correctly report among those people without diabetes. The elderly were more prone to be aware of the diabetes, while less inclined to report their absence of diabetes. The false reporting and false negative reporting of diabetes went up significantly with age.

We draw three lessons from our results. First, self-reported data underestimated the disease burden of hypertension and diabetes, and the underestimation of diabetes was even greater. Adding objective measurements to social survey could improve data accuracy and allow better understanding of socioeconomic inequalities in health. We underline the need to supplement subjective health data with comprehensive and reliable biomedical measurements. Biomarkers are more valid measures of physiological function “under the skin”, meaning biosocial approaches to enhance the importance of social factors in the biomedical process and to intervene in social conditions that cause inequity and avoidable inequity will become increasingly important [19]. Second, we found that there was a big discrepancy between self-reported diseases and biomedical tests on hypertension and diabetes in China. China is undergoing rapid population aging, accompanied by non-communicable and chronic conditions. Although inclusion of biological and anthropometric measures of health in surveys expands the possibilities for biomarkers and social construction, underdiagnosis of disease is still common in China. Considering the underdiagnosis of disease, Chinese government needs to increase the awareness of disease and reassess the burden of disease. Third, there is an urgent need to provide basic health education and physical examination to citizens, and promote the use of healthcare to lower the incidence and unawareness of disease in China.

The challenge of identifying causal effects remains universal in most science research, including social stratification and health research. In this regard, longitudinal data with biomarker or genetics are especially useful for sorting out causal effects. For example, having baseline biomarker measures prior to some social exposure or self-assessment enables researchers to explore whether and to what extent age trajectories of self-reported health, biomarkers and their discrepancy, depended on the sociodemographic characteristics. A drawback of the paper is that we used cross-sectional data, which limits causal inferences of sociodemographic on the discrepancy. Another limitation is that biomedical measurement is imperfect. The most important source of confounding is the failure to identify factors that may alter the measurement of the biomarker, such as metabolic factors. Future work could also consider additional data sources and repeated multiple biomarker measurements to complement survey data.

## Conclusions

The prevalence of hypertension and diabetes are increasing over age in China, with many old people remaining undiagnosed. Self-reported hypertension and diabetes showed low sensitivity (73.24 and 49.21%, respectively), but high specificity (93.61 and 98.05%, respectively). False positive reporting of hypertension and diabetes were 3.97 and 1.67%, while false negative reports were extremely high at 10.14 and 7.38%. Educational attainment, *hukou*, age and gender affected both the group-specific error and the overall error of reporting hypertension and diabetes, but there were some differences in the magnitude and directions. As we know, this is the first report of undiagnosed hypertension and diabetes by using four indicators and evaluating sociodemographic characteristics that were correlated with misreporting in China, and the results confirm self-reported conditions underestimate the disease burden. Adding objective measurements into social survey could improve data accuracy and allow better understanding of socioeconomic inequalities in health.

## Data Availability

The datasets generated and/or analyzed during the study are publicly available. http://charls.pku.edu.cn

## Abbreviations

HbA1c: Glycated hemoglobin
FPG: Fasting plasma glucose
CHARLS: China health and retirement longitudinal study
DALY: Disability-Adjusted Life Year

## Footnotes

1 The distribution was very similar and no bias was found.

2 Each respondent who did not choose hypertension, was continued to ask “do you know whether you have hypertension by yourself”.

3 In order to assess the differences in communities and provinces, we also adopted random intercept model, the results showed that variations between communities and provinces were not statistically significant.
